# COVID-19 Impact and Vaccination Willingness among Romanian Patients with Autoimmune/Immune-Mediated Diseases

**DOI:** 10.3390/healthcare9121707

**Published:** 2021-12-08

**Authors:** Larisa Pinte, Simona Caraiola, Daniel Vasile Balaban, Camelia Badea, Diana Mazilu, Georgeta Daniela Ionescu, Maria-Ilinca Iosub, Elena-Sabina Bălan, Florentina Negoi, Bianca Dumitrescu, Bogdan Mateescu, Ruxandra Ionescu, Magda Ileana Parvu, Cristian Baicus

**Affiliations:** 1 Faculty of Medicine, Carol Davila University of Medicine and Pharmacy, 050474 Bucharest, Romania; scaraiola@yahoo.com (S.C.); vbalaban@yahoo.com (D.V.B.); cameliabadea72@yahoo.com (C.B.); dimazilu@yahoo.com (D.M.); bia1mar@yahoo.com (B.D.); bogmateescu@gmail.com (B.M.); ruxandraionescu1@gmail.com (R.I.); cbaicus@gmail.com (C.B.); 2 Internal Medicine, Rheumatology and Gastroenterology Departments, Colentina Clinical Hospital, 072202 Bucharest, Romania; ionescu_georgiana93@yahoo.com (G.D.I.); ilinca.iosub@gmail.com (M.-I.I.); sabina.balan05@gmail.com (E.-S.B.); flori.negoi28@gmail.com (F.N.); parvumagda@yahoo.com (M.I.P.); 3 Gastroenterology and Internal Medicine Departments, “Dr. Carol Davila” Central Military Emergency University Hospital, 01082 Bucharest, Romania; 4 Rheumatology and Internal Medicine, Sf. Maria Clinical Hospital, 011172 Bucharest, Romania; 5 Rheumatology Department, Ion Stoia Clinical Centre of Rheumatic Diseases, 030167 Bucharest, Romania

**Keywords:** autoimmune, immune mediated, rheumatic diseases, COVID-19, SARS-CoV2, vaccine willingness, survey

## Abstract

Background: During the COVID-19 pandemic, patients with immune diseases are a vulnerable population. We aimed to evaluate their access to medical care, as well as their awareness and willingness to obtain the vaccine after a year of the SARS-CoV-2 pandemic. Methods: A cross-sectional, multicenter study was conducted on a questionnaire basis, handled both online as well as in person. Results: 651 patients with autoimmune or immune mediated diseases were enrolled. More than half (339/641 [53%]) reported difficulties in obtaining medical care throughout the pandemic and 135/651 ([21%]) of them were confirmed with COVID-19; 442/651, ([68%]) expressed their willingness to be vaccinated against SARS-CoV-2. The factors associated with an increased probability of vaccination were the male gender (OR = 2.01, CI95% 1.2–3.7, *p* = 0.001), the patient’s opinion that she/he was well informed (OR = 3.2, CI 95% 2.1–6.01, *p* < 0.001), physician’s advice (OR = 2.1, CI 95% 1.3–3.5, *p* < 0.001), and flu vaccination in the past (OR = 1.5, CI 95% 1.1–2.3, *p* < 0.001), while those associated with a decreased probability of vaccination were COVID-19 disease in the past medical history (OR = 0.7, CI 95% 0.3-0.95, *p* = 0.02), and the opinion that patients with autoimmune diseases are at increased risk for adverse reactions (OR = 0.7, CI95% 0.53–0.89, *p* = 0.001). Conclusions: Given the fact that considering themselves informed regarding vaccination is the most important factor in order to be immunized against SARS-CoV-2, effective information campaigns would substantially increase willingness.

## 1. Introduction

Severe acute respiratory syndrome coronavirus 2 (SARS-CoV-2) was identified in December 2019 in China’s Hubei Province and soon spread around the world, causing a pandemic with significant impact on the medical system.

Until now, with 4.5 million deaths, COVID-19 ranks eighth in history’s most deadly pandemics [1].

Patients with immune diseases, generally considered a vulnerable population have been facing the risk of anxiety (57.3%) and depression (45.9%), while lacking information regarding the possible impact of SARS-CoV-2 infection on their pathology (45.6%) [2]. An EULAR (European Alliance of Associations for Rheumatology) guided survey highlighted that 82% of the physicians involved in treating patients with autoimmune/immune- mediated diseases cancelled or postponed face-to-face visits, offering in return remote evaluations for both new patients and follow-up visits [3].

With a higher risk of COVID-19 compared to the general population [4] and worse outcomes, mostly associated with corticosteroid and immunosuppressive therapy use on a regular basis [5,6], patients with immune diseases remain a vulnerable population in need of careful monitoring.

As such, we aimed to evaluate their course in terms of access to medical care and COVID-19 management, as well as their awareness and willingness to get the vaccine after a year of the SARS-CoV-2 pandemic.

## 2. Materials and Methods

We performed a cross-sectional observational study conducted on the basis of a questionnaire, open for response from 5 February–7 May 2021, comprising three sections. The first section gathered demographic data and information regarding diagnosis, background treatment, comorbidities, as well as access to medical care during the pandemic. The second section assessed features regarding COVID-19 disease in these patients, while the third section evaluated their perspective, basic knowledge and willingness to be immunized against SARS-CoV-2 virus.

The study was conducted in accordance with CHERRIES recommendations [7]. Close-ended answer choices were applied in most of the questions. The full questionnaire is available as Supplementary Material 1. 

The survey was handled both online via Survey Monkey link posted on social media in patient groups with immune disorders, as well as in person during regular face-to-face evaluations, in a multicentre collaboration with physicians involved in treating patients with autoimmune/immune- mediated diseases. Patients with blunted or altered immunity related to oncological diseases or post-transplant were not included.

In order to assess face validity, three rheumatologists (CB, SC and FN), and the administrator of the patients group with autoimmune diseases (RL) reviewed the questions and considered them relevant for the objective of the study. The questionnaire was administered to 20 patients for pilot testing and as a result 12 questions were either modified, or deleted. The revised version of the survey was administered both on paper and online to 10 participants in order to evaluate the repeatability. We calculated the intra-class correlation coefficient (ICC) of the items, which was between 0.64 and 1.

The subjects who completed the survey online could revise their answers prior submission.

The study was conducted in accordance with the General Data Protection Regulation of the European Union, applied from 25 May 2018. Subjects were asked to provide their telephone number as identification ID and follow-up contact.

Patients in whom the survey was applied during face-to-face evaluations were enrolled after completing the informed consent form. For respondents who accessed the survey online, consent to participate in the research was given by reading the introductory paragraph and opening the questionnaire items.

The online survey was distributed in closed patient groups affiliated to patient associations, and membership usually required a control mechanism imposed by the administrator.

In addition, in order to confirm that the respondents were actually patients, each subject was contacted by telephone after completing the survey. Patients without autoimmune/immune- mediated diseases were excluded after the telephone evaluation.

Only two members of the study team (CB, LP) had access to the entire database, guaranteeing the security of the data. Doctors who enrolled patients during face-to-face visits had access only to the information provided by the patients they enrolled.

Data analysis was performed using IBM SPSS Statistics for Windows, version 20 (IBM Corp., Armonk, NY, USA) and Microsoft Excel 2018 (Microsoft Corporation, Redmond, WA, USA). Categorial variables were summarized as number (percentage) and analyzed with the Chi square test, while the numeric variables without normal distribution were summarized as median (min, max), and compared by the Mann–Whitney U test. The variables which were associated with a *p* ≤ 0.1 with the outcome (vaccination) were further introduced in multivariable models (binary logistic regression). *p* value < 0.05 was considered statistically significant.

## 3. Results

### Characteristics of the Respondents

**Figure 1:**
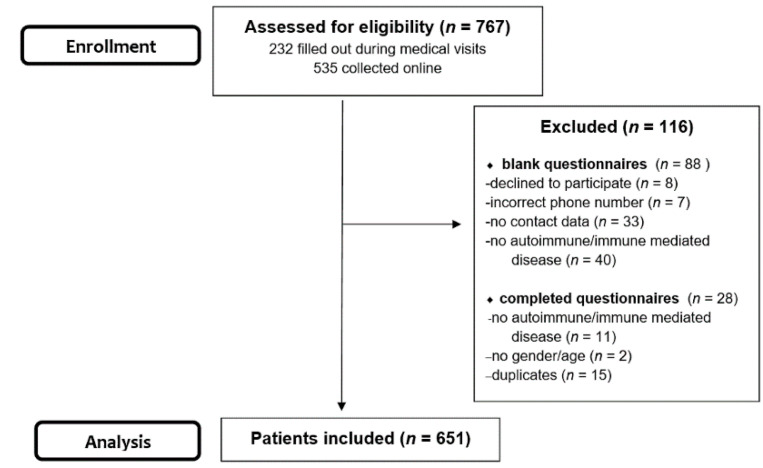
Patient’s selection flow diagram.

A total of 767 answers were gathered from both sources, 232 surveys were filled out during medical visits, while the rest were collected online. Responders who submitted blank or partially incomplete questionnaires were contacted by phone (when number provided) in order to complete the missing data. After the exclusion of patients without autoimmune/immune- mediated diseases and duplicates, 651 surveys were considered eligible for the study. (Figure 1)

Overall, we identified 651 patients with autoimmune/immune- mediated diseases. The sample had a median age of 49 (minimum = 19, maximum = 88) years, and 546 (84%) were women and contained 411 with rheumatic and musculoskeletal (RMD) diseases.

Most of the responders (511/651 [79%]) were non-health care professionals, while the rest were also either health care workers (doctors 43/651 [6%] or nurses 29/651 [5%]) or had connection with the medical system (68/651 [10%]).

Common pathologies identified were autoimmune thyroid disease (140 [22%]), rheumatoid arthritis (104 [16%]), systemic lupus erythematosus (SLE) (99 [15%]) Sjögren’s syndrome (80 [12%]), ankylosing spondylitis (69 [11%]), psoriatic arthritis (55 [8%]), inflammatory bowel disease (IBD) (44 [7%]), systemic sclerosis (SSc)/limited scleroderma (31 [5%]), myasthenia Gravis (30 [5%]), antiphospholipid syndrome (AFLS) (26 [4%]), multiple sclerosis (25 [4%]), hepatic autoimmunity (21 [3%]), systemic vasculitis (20 [3%]), celiac disease (20 [3%]), dermatomyositis/polymyositis (12 [2%]), as well as other immune mediated disorders listed in Supplementary Material 1-Table S1.

Except for a minority of the patients (29/651 [4%]) who associated three or more autoimmunities, the rest reported one (513/651 [79%]) or two (109/651 [17%]) immune diseases.

In addition to conventional synthetic DMARDs (disease-modifying anti-rheumatic drugs), such as hydroxychloroquine (HCQ) (137 [21%]), methotrexate (72 [11%]), sulfasalazine (50 [8%]) and leflunomide (21 [3%]), the patients were also taking glucocorticoids (144 [22%]), biologic DMARDs (133 [21%]) as well as other immunosuppressive drugs such as azathioprine (46 [7%]), mycophenolate mofetil (18 [3%]) and cyclophosphamide (2 [0.3%]).

**Figure 2:**
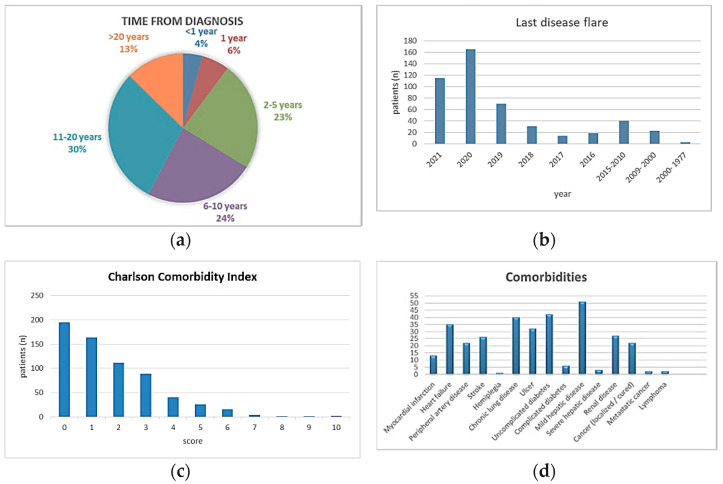
(**a**) Diagnosis of immune disease; (**b**) year of the last flare; (**c**) Charlson comorbidity index; (**d**) comorbidities reported.

More than half of the responders (442/637 [69%]) had a diagnosis of autoimmune/immune- mediated disease for more than five years, while 4% (27/637) of them were diagnosed in the past year (Figure 2a). 

When asked to mention “the last date of their disease aggravation (flare)”, only 75% (481/651) of them were able to answer the question. More than half of the patients (280/481 [58%]) reported having a flare since the beginning of 2020, and 9% (26/280) of them reported the flare after COVID-19 disease. (Figure 2b)

As presented in Figure 2c,d, half of the patients (324/651 [50%]) had at least one additional pathology. Also, 11% of the subjects reported pulmonary damage as a consequence of their autoimmune/immune- mediated disease.

A. Access to medical care during the pandemic

More than half of the responders (339/641 [53%]) reported difficulties in obtaining medical care throughout the pandemic, mostly because their regular hospital became a COVID-19 support facility (126/339 [37%]), were afraid of contracting the virus (120/339 [35%]), or considered the appointments difficult to obtain (41/339 [12%]).

The rest of the patients [47%], stated that they either did not require medical care (123/641 [19%]) or were successfully assessed by their attending physician at its private practice and using telemedicine (141/641 [22%]), while 6% (41/641) of them were addressed to primary care physicians for treatment continuance and monitoring.

In addition, only a small part of the subjects responded that their hospital continued its regular activity (80/641 [12%]), while (24/641 [4%]) of them were forced to change their doctor.

B. SARS-CoV-2 infection in subjects with autoimmune and immune-mediated diseases

Although only 26% (167/651) of the responders had symptoms resembling COVID-19, half (331/651 [51%]) of them were tested at least once of their own free will. Four percent (23/651 [4%]) avoided testing despite having symptoms as they were afraid of mandatory hospital admission.

A total of 135 subjects (135/651 [21%]), 114 females and 21 males, median age 47 (minimum = 19, maximum = 84), were confirmed with the infection using RT-PCR (96/135), rapid antigen test (27/135) or both (12/135).

Almost a quarter (33/135 [24%]) of the positive patients denied having contact with infected individuals.

Autoimmune thyroiditis (24 [13%]), SLE (22 [12%]), rheumatoid arthritis (16 [9%]), Sjogren syndrome (16 [9%]), psoriasis (14 [8%]), IBD (14 [8%]), spondylo arthritis (13 [7%]), SSc/limited scleroderma (8 [4%]), myasthenia gravis (7 [4%]) and AFLS (6 [3%]) were the most frequent autoimmune disease entities from this group. (Supplementary Material 1-Table S1).

Twenty-eight percent of the patients (38/135 [28%]) were not receiving treatment for their autoimmune disease, and they did not require treatment during the SARS-CoV-2 infection, either.

The rest were receiving corticosteroids (26 [19%]), conventional synthetic DMARDs, such as HCQ (29 [21%]), methotrexate (13 [10%]), leflunomide (9 [6%]), sulfasalazine (8 [6%]), as well as azathioprine (12 [9%]), mycophenolate mofetil (5 [4%]) and biologic DMARDs (31 [23%]).

Most of the patients enrolled had mild COVID-19 and the top symptoms reported were anosmia (79/135), dry cough (76/135), general weakness (74/135), ageusia (65/135), fever (60/135), headache (56/135), myalgia (55/135), chills (50/135), sore throat (40/135), diarrhea (31/135), nausea/vomiting (34/135), dyspnea only at exertion (25/135) or at rest (19/135), rhinorrhea (18/135), fatigue (17/135), productive cough (14/135), arthralgia (5/135), sweating (3/135), loss of appetite (2/135) as well as memory disorders (1/135) and dizziness (1/135).

Only 19% (26/135) of the patients were admitted to the hospital. During their stay in the hospital 11/26 received oxigenotherapy, of whom four were managed in the intensive care unit, while 6/26 considered that hospitalisation was not required at all. The rest were monitored by their treating physician or general practitioner as outpatients (62/135 [46%]), or handled COVID-19 on their own (47/135 [35%]).

When asked about the pulmonary involvement secondary to SARS-CoV-2 infection, only 26 patients from 127 responders acknowledged having a documented viral pneumonia, most of them mild (17/26) or moderate (6/26).

Frequently, they were prescribed antibiotics (50/135), anticoagulants (38/135), symptomatic treatment (38/135) or vitamins (35/135), while tocilizumab (3/135), anakinra (3/135), remdesivir (8/135), lopinavir/ritonavir (7/135), favipiravir (5/135), umifenovir (1/135) and convalescent plasma (1/135) were administered in a smaller number of subjects. Corticosteroids and HCQ were prescribed exclusively for COVID-19 in 22 and 7 patients, respectively.

Regular background treatment was administered without changes in 64% (62/97) of the patients, adjusted in 10% (10/97) and discontinued in 26% (25/97) of them.

C. Perspective, basic knowledge and willingness to be immunized against SARS-CoV-2

Eighty five percent of the participants (546/640) considered themselves informed regarding the national program of immunization.

The most common sources of information were the media (71%), treating physician (34%), general practitioner (23%) and patient associations (10%). Information obtained from a friend/colleague/work environment (6%), medical literature (3%), family (2%), vaccination platform (2%) or another patient (1%) were less mentioned.

Most of the responders (442/651, [68%]) expressed their willingness to be vaccinated against SARS-CoV-2. A total of 250 (60.7%) patients received the first dose of the COVID-19 vaccine before enrolment. The patients enrolled online had an increased willingness of vaccination (70% vs. 30%, *p* < 0.001) and were more likely to decide to be vaccinated (43% vs 35%, *p* = 0.06) compared with those enrolled during face-to-face evaluations.

Reasons for refusing the vaccine were fear of side effects (118/203), lack of confidence in the promoted effect (21/203), doctor’s contraindication (18/203) as well as the history of COVID-19 (18/203), lack of contact with infected subjects (15/203) or insufficient studies involving patients with immune pathologies (3/203). Only a few of them (21/645) were undecided as to whether to be vaccinated or not.

Males were more inclined to be vaccinated (OR = 1.8, CI 1.1–3, *p* = 0.015), while subjects who completed the survey during face-to-face evaluations were less inclined to be vaccinated (OR = 0.5, CI 0.36–0.71, *p* < 0.001). Although there was a significant difference regarding gender between the patients who completed the questionnaire online or face-to-face—males completed the survey mostly during hospital evaluations (OR = 3, CI 1.9-4.44, *p* < 0.001), there were no differences regarding age (*p* = 0.09), or Charlson index (*p* = 0.066).

In logistic regression, the factors associated with an increased probability of vaccination were the male gender (OR = 2.01, CI95% 1.2–3.7, *p* = 0.001), the patient’s opinion that she/he was well informed (OR = 3.2, CI95% 2.1–6.01, *p* < 0.001), physician’s advice (OR = 2.1, CI95% 1.3–3.5, *p* < 0.001), and flu vaccination in the past (OR = 1.5, CI95% 1.1–2.3, *p* < 0.001), while those associated with a decreased probability of vaccination were completing the survey during face-to-face evaluations (OR = 0.44, CI95% 0.31–0.67, *p* < 0.001), having COVID-19 disease in their past medical history (OR = 0.7, CI95% 0.34–0.95, *p* = 0.02), or the opinion that patients with autoimmune diseases are at increased risk for adverse reactions (OR = 0.7, CI95% 0.53–0.89, *p* = 0.001).

In terms of adverse reactions, 83% (524/635) of the responders considered themselves informed and the most mentioned source of information was the media (284/635), followed by medical articles (121/635), their treating physician (83/635) and general practitioner (52/635). The rest of the patients, either did not associate the vaccine with potential side effects (39/635) or were not interested in being informed (32/635) or denied access to information sources (40/635).

When asked if they considered themselves to be at greater risk of developing post immunization adverse reactions due to their immunological pattern, 51% (320/631) of them gave a positive response, while the rest either denied (144/631 [23%]) having additional risk compared to the general population or were unable to evaluate their risk (167/631 [26%]).

However, more than half (184/320 [58%]) of those who considered that the vaccine might have an impact on their disease activity, were willing to take the risk.

More than half of the responders (450/635 [71%]) approached their treating physician for vaccine-related medical advice, while the rest did not consider it necessary to discuss this topic (116/635 [18%]) or could not reach their doctor (69/635 [11%]). Of those seeking medical guidance, it was recommended to 63% (282/450) to receive the vaccination and only 6% (26/450) were advised to avoid immunization due to their autoimmune status.

Regarding influenza vaccination of the 624 responders, 59% (366/624) had never been vaccinated, 15% (93/624) had the vaccine annually, 14% (84/624) usually were vaccinated but not every year, while 5% of them (31/624) were vaccinated frequently. In addition, 42 patients decided to do it this year, regardless of their previous hesitancy.

## 4. Discussion

In an attempt to limit COVID-19 transmission, face-to-face evaluations for chronic patients were reserved for severe cases only. The rest were assessed by telemedicine, a satisfactory option on both sides at least in patients with mild forms of the disease [8,9,10].

Since the first months of the pandemic, Romanian health policy imposed the conversion of various hospitals exclusively into COVID-19 facilities, destined only for the care of SARS-CoV-2 infected patients, including asymptomatic ones, explaining the difficult access to medical care among the responders. In addition, this decision led treating physicians to feel that “chronic rheumatic patients were left aside, while caring mostly for COVID-19 patients” [11].

In addition to limited access to health care, the use of anti-malarials worldwide as primary prophylaxis and initially as treatment for SARS-CoV-2 infection caused shortages of hydroxychloroquine provoking distress especially among patients with SLE, in whom treatment efficacy has been already established in studies [12]. Regardless of its properties, 29 of the 137 patients receiving HCQ as a background treatment were diagnosed with COVID-19.

A recently published meta-analysis, gathering 319,025 subjects with rheumatic diseases, showed that the prevalence of COVID-19 in these individuals is low (0.011, 95% CI: 0.005–0.025), and although it remains significantly higher compared to the general population (OR = 1.53, 95% CI: 1.24–1.88, *p* = 0.0001), the risk of death is similar (OR = 1.29, 95% CI 0.84–1.97, *p* = 0.248) [4,13].

Although studies showed that patients with rheumatic diseases have a higher risk of contracting the virus [4] and glucocorticoid use along with a high dose of immunosuppressors [14] raised the chance of unfavorable outcomes [15,16], most of our responders had mild COVID-19, as only 19% of them required hospitalization.

It has been shown that higher age [17], cardiovascular, pulmonary and chronic kidney disease were independent risk factors for hospital admission [13,18], while coexisting immune pathologies and increased disease activity raised the odds of severe COVID-19 [19] and death [20].

A plausible explanation for the mild forms of COVID-19 reported in our sample was the fact that most of the responders had a single autoimmune disorder (108/135 [80%]) and a Charlson Comorbidity Index lower or equal to 2 (102/135 [76%]). In addition, pre-existing pulmonary damage did not predispose to a severe form of COVID-19.

The national vaccination program was launched in Romania on 27 December 2020, using the BNT162b2 mRNA Covid-19 Vaccine (Comirnaty, Pfizer). The immunization campaign was planned in three consecutive rounds—vaccine was first available for health care workers, followed by chronic disease patients and individuals aged over 65 in the second round and the general population in the third round. Vaccination centers were set up within medical units and public institutions in local communities. mRNA-1273 vaccine (Spikevax, Moderna) was available since 4 February, AZD1222/ChAdOx1 (Vaxzevria, AstraZeneca) since 15 February and JNJ-78436735 (Janssen, Johnson and Johnson) since 4 May 2021. Patients with autoimmune diseases were eligible for vaccination in the second round of the vaccination campaign, which overlapped with the third wave of COVID-19 in Romania and with social restrictions and lockdown measures in place [21].

The VAXICOV study, a worldwide online questionnaire, conducted in 56 countries, mostly France, United Kingdom, Chile, United States of America, Venezuela, Spain, Mexico and Argentina, which collected data from 265 healthcare professionals and 1266 patients with autoimmune and rheumatic diseases, showed that patients with autoimmune diseases were more afraid of being infected compared to healthcare professionals, but their willingness to get vaccinated against SARS-CoV-2 remained moderate [22]. However the immunization rate in our cohort was higher than in the general population [23].

Given the fact that one of the most important causes of vaccine hesitancy in patients with autoimmune diseases is their concern towards adverse reactions and the increase in their autoimmune disease activity [24], we advocate that a high degree of information (media included) would substantially increase their willingness.

As such, besides consulting with their treating physician, which increased vaccination willingness up to 20% [24], another promising strategy could be involving healthcare students in the vaccination campaigns [25].

However, on the verge of the fourth wave, achieving the target level of herd immunity via vaccination remains unlikely in Romania, as until 11 September 2021 only 27% of the general population was fully vaccinated against COVID-19 [23].

Given the fact that the survey was designed for patients with rare disorders, a strength of the study is the large sample size we were able to gather.

Also it is the first study to highlight features regarding SARS-CoV-2 infection in this category of patients. The number of COVID-19 patients enrolled is similar to what Romania reported in the EULAR—COVID-19 [26] and SECURE-IBD [27] databases at the time the study closed to recruitment.

The patients recruited during face-to-face evaluations were less willing to be vaccinated, even after adjusting for gender, which was different between the sources of recruitment (a significantly higher proportion of women recruited online), which means that there were also other differences between these groups of patients (probably education, but unfortunately this was not measured by our questionnaire).

The main weakness of this study is the fact that it was a convenience sample, and probably not very representative for all of this group. Excepting the patients recruited in the clinics, the other patients were patients with internet access, and/or members of patient’s associations, and therefore patients with a certain level of education.

Another limitation of our study might be the selection bias given the fact that most of the responders enrolled were already considering vaccination. Therefore, our findings might not apply to the entire population of patients with autoimmune /immune-mediated diseases.

A substantial limitation of the study concerns SARS-CoV-2 infection. Even if based on our results it might appear appropriate to assume that their course of the disease was a mild one, only the patients who survived could have completed the questionnaire, therefore we cannot conclude concerning the survival. We did not have access to population registries in order to accurately evaluate the death rate among COVID-19 patients with autoimmune diseases/immune-mediated diseases. In addition, studies published in Romania until now listed among the most common comorbidities associated with COVID-19 deaths diabetes, chronic renal disease, hypertension and obesity [28,29], but none of them mentioned autoimmune or immune-mediated diseases.

## 5. Conclusions

Even though access to health care was obstructed during the pandemic, most of the patients with autoimmune /immune-mediated pathologies managed to find a suitable way to monitor their disease. Also, it appears that while contracting the virus their course of the disease was mostly a mild one. Factors that contributed to their decision to obtain the COVID vaccine were considering themselves informed regarding vaccination, doctor’s advice, and the habit of getting the flu shot. We advocate that effective information campaigns would substantially increase their willingness to be immunized against SARS-CoV-2.

## Data Availability

Data used to support the findings of this study are included within the article and in the Supplementary Material 1. Any additional data are available from the corresponding author upon request.

## Supplementary Materials

The following are available online at https://www.mdpi.com/article/10.3390/healthcare9121707/s1, Supplementary Material 1: Full questionnaire, Table S1: Autoimmune/immune mediated diseases reported.
