# Supplementary material for: COVID-19 Impact and Vaccination Willingness among Romanian Patients with Autoimmune/Immune-Mediated Diseases
Source: Healthcare (Basel). 2021 Dec 8;9(12):1707. doi: 10.3390/healthcare9121707 (PMC8700907; doi:10.3390/healthcare9121707)
Supplement: Supplementary file 1 [file healthcare-09-01707-s001.zip › healthcare-1407939-supplementary.pdf]

Questionnaire

General Data:
Gender (Male/Female/Other)
Age
Do you work in the medical field? (No, contact with the medical field, doctor, nurse)
What Autoimmune/immune mediated disease do you have? (Multiple-choice question)
What is your background therapy? (Multiple-choice question)
Do you take Biologic DMARDs? (Yes/No)
When was your immune disorder diagnosed? (Open-ended question)
When was the last date of the disease aggravation (flare)? (Open-ended question)
Do you have pulmonary damage as a consequence of your immune disease? (Yes/No)
What other pathologies do you have? (Multiple-choice question)

A. Access to medical care during the pandemic

Have you encountered difficulties in accessing medical care since the beginning of the pandemic? (Yes/No)
If yes, why? (postponed visits for fear of infection/ my regular hospital became a COVID-19 support facility/ the appointments were difficult to obtain/ other response)
If no, how? (did not require medical care/ assessed by the attending physician at their private practice and using telemedicine/ addressed to primary care physicians/ the hospital continued its regular activity)

B. SARS-CoV-2 infection in subjects with autoimmune and immune-mediated diseases

Did you have symptoms resembling COVID-19? (Yes/No)
Did you get tested for COVID-19? (Yes, the result was positive-was in contact with a positive person/Yes, the result was positive-without contact with a positive person/Yes, the result was negative/No, because I had no contact with positive people and nor symptoms/No, because I had no symptoms, although I had contact with positive people/No, although I had symptoms, because I was afraid of hospitalization)
Have you tested positive for COVID19? (Yes/No)
If yes, by which type of test? (RT-PCR/rapid test)
Mention the date of COVID-19 diagnosis. (Open-ended question)
What symptoms did you have? (Multiple-choice question)
How was COVID-19 managed? (hospitalised, but I don't think I needed to/ hospitalised, because that was the law/ hospitalised (I needed to)/monitored at home/not monitored)
Did you have pulmonary involvement secondary to SARS-CoV-2 infection? (No/ Not investigated/ Yes- mention the degree of the involvement)
If admitted to the hospital, did you require oxigenotherapy? (Yes/No)

Did you require ICU admission? (Yes/No)
Mention the length of hospitalisation. (Open-ended question)
What treatment was recommended for COVID-19? (Multiple-choice question)
How was the treatment for your immune disease managed during the infection? (continued without changes/ doses have been adjusted/ immunosuppressive therapy has been stopped)
What was the evolution of the immune pathology during the pandemic? (Stationary/ it got worse-have not had COVID/ got worse after COVID)

C. Perspective, basic knowledge and willingness to be immunized against SARS-CoV-2

Do you consider yourself informed regarding the national SARS-CoV-2 immunization program? (Yes/No)
Where did you get the information about the vaccination program? (Multiple-choice question)
Do you want to get vaccinated against SARS-CoV-2? (Yes/No, because I don't think I'll get in touch with infected people/ No, because I had COVID-19/No, because I'm afraid of possible side effects/ No, because I don't think it works/ No, because the doctor advised me not to get vaccinated)
Do you know the possible side effects of the SARS-CoV-2 vaccine? (Semi-structured, multiple-choice question)
Do you consider yourself at higher risk of developing side effects given your immune pathology? (No, I have no higher risk than the rest of the population/ Yes, but I will take the risk/ Yes and I don't want to take the risk/ I don't know if I'm at risk or not)
Have you talked to your doctor about SARS-CoV-2 vaccination? (Semi-structured, multiple-choice question)
What is your attitude towards the FLU vaccine? (vaccinated annually/ never vaccinated/ usually got vaccined but not every year/frequently/ vaccinated this year)
Did you get the Coronavirus Vaccine? (Yes/No)

Table S1. The incidence of COVID-19 among the autoimmune and imune mediated diseases reported *.

Immune disease	Covid-19 patients positive (*n* = 135)	Covid-19 negative (*n* = 516)	*p* value
**Rheumatic Diseases and Musculoskeletal Conditions**			
Rheumatoid arthritis	16 (11.9%)	88 (17.1%)	0.149
Systemic lupus erythematosus	22 (16.3%)	77 (14.9%)	0.687
Sjögren's syndrome/SICCA	16 (11.9%)	64 (12.4%)	1
Ankylosing spondylitis	13 (9.6%)	56 (10.9%)	0.755
Psoriatic arthritis/psoriasis	14 (10.4%)	41 (7.9%)	0.385
Systemic sclerosis/limited scleroderma	8 (5.9%)	23 (4.5%)	0.496
Antiphospholipid syndrome	6 (4.4%)	20 (3.9%)	0.805
Dermatomyositis/polymyositis	1 (0.7%)	11 (2.1%)	0.476
Mixed connective tissue disease	2 (1.5%)	8 (1.6%)	1
Behçet's disease	0 (0%)	3 (0.6%)	1
Granulomatosis with polyangiitis	4 (3%)	1 (0.2%)	**0.007**
Microscopic polyangiitis	1 (0.7%)	2 (0.4%)	0.503
Giant cell arteritis	2 (1.5%)	3 (0.6%)	0.278
Takayasu arteritis	1 (0.7%)	0 (0%)	0.207
Polyarteritis nodosa	0 (0%)	1 (0.2%)	1
Henoch–Schönlein purpura	0 (0%)	1 (0.2%)	1
Cryoglobulinemia	0 (0%)	1 (0.2%)	1
**Gastroenterological and hepatic diseases**			
Inflammatory bowel disease	14 (10.4%)	30 (5.8%)	0.081
Celiac disease	4 (3%)	16 (3.1%)	1
Primary biliary cholangitis	3 (2.2%)	6 (1.2%)	0.402
Autoimmune hepatitis	2 (1.5%)	10 (1.9%)	1
**Neurological diseases**			
Myasthenia gravis	7 (5.2%)	23 (4.5%)	0.651
Multiple sclerosis	1 (0.7%)	24 (4.7%)	0.041
Autoimmune encephalomyelitis	1 (0.7%)	1 (0.2%)	0.372
**Hematological diseases**			
Hemolytic anemia	1 (0.7%)	1 (0.2)	0.372
Paroxysmal Nocturnal Hemoglobinuria	1 (0.7%)	0 (0.%)	0.207
Autoimmune thrombocytopenia	0 (0%)	3 (0.6%)	1
**Cutaneous diseases**			
Vitiligo	2 (1.5%)	8 (1.6%)	1
Cutaneous lupus	1 (0.7%)	1 (0.2%)	0.372
Cutaneous vasculitis	1 (0.7%)	3 (0.6%)	1
Pemphigus	0 (0%)	1 (0.2%)	1
**Other immune mediated diseases**			
Autoimmune thyroid disease	24 (17.8%)	116 (22.5%)	0.289
Sarcoidosis	4 (3%)	4 (0.8%)	0.062
Type 1 diabetes mellitus	1 (0.7%)	2 (0.4%)	0.503
Hyper IgD syndrome	0 (0%)	1 (0.2%)	1

* Participants may report more than one autoimmune/immune mediated disease.
